# Effects of Alkaline Mineral Complex Water Supplementation in the Diet on the Oxidative Stress, Immunity and Health of Lactating Cows

**DOI:** 10.3390/ani16182915

**Published:** 2026-09-16

**Authors:** Zhanshan Yang, Yang Zou, Erdan Wang, Shengli Li

**Affiliations:** 1 Shenzhen Freedom Trade Co., Ltd., Fengye Dasha 4L, No. 1088 Nanshan Street, Nanshan District, Shenzhen 518000, China; 2 Independent Researcher, Beijing 100194, China; 3 State Key Laboratory of Animal Nutrition, Beijing Engineering Technology Research Center of Raw Milk Quality and Safety Control, College of Animal Science and Technology, China Agricultural University, No. 2 Yuanmingyuan West Road, Haidian District, Beijing 100193, China; 4 College of Animal Science and Technology, College of Veterinary Medicine, Zhejiang Agricultural and Forestry University, No. 666 Wusu Street, Lin’an District, Hangzhou 311300, China

**Keywords:** mid-lactation cow, alkaline mineral complex water, oxidative stress, immunity

## Abstract

Mid-lactation represents a critical production phase for high-yield dairy cows, where nutrient imbalance can compromise animal health and farm profitability. This study investigated the effects of dietary alkaline mineral complex (AMC)–water supplementation on milk yield, blood biochemistry, oxidative stress, immunity, hormone secretion and ion status in mid-lactation dairy cows. A total of 120 high-yield mid-lactation Holstein cows were stratified into three milk yield subgroups (EHYG, HYG, MHYG) based on pre-experimental milk yield records. Within each yield stratum, 20 cows were randomly assigned to either the control group or the AMC-supplemented group, resulting in three control subgroups and three AMC-supplemented subgroups (*n* = 20 per subgroup). Controls received a basal diet, while cows in experimental groups received the basal diet supplemented with diluted AMC concentrate solution (1:4 concentrate to water, *v*/*v*) for 90 d. Blood samples were collected on days 0, 15, 30, 45, 60 and 90, and milk samples were obtained on days 0, 30, 60 and 90 to evaluate the production performance, stress and immune status. Dietary AMC supplementation reduced plasma NEFA (*p* < 0.01) and BHBA concentrations (*p* < 0.05). For hormone, oxidative-stress and immune indices, significant differences were detected only at discrete individual sampling time points, with no consistent overall treatment effects across the entire experimental period. No significant overall treatment effect of AMC supplementation was observed on the milk yield. These results provide preliminary reference data for alkaline mineral complex additive application in practical mid-lactation dairy production.

## Simple Summary

Mid-lactation is an important stage affecting the production performance and health of dairy cows. Imbalanced nutrition may impair the production performance and economic returns in dairy farming. To explore nutritional regulation strategies for mid-lactation dairy cows, this study evaluated dietary supplementation with alkaline mineral complex (AMC) water. A total of 120 high-yield dairy cows were assigned to control and experimental groups. The control group received a basal diet, whereas the experimental group was supplied with the basal diet plus diluted AMC solution for 90 d. Blood and milk samples were collected periodically to determine the milk yield, stress status, immunity, and hormone and ion profiles. AMC supplementation produced partial beneficial effects on physiological indexes of mid-lactation dairy cows. This work offers a practical technical reference for healthy dairy cow breeding and sustainable development of the dairy industry.

## 1. Introduction

The mid-lactation stage of dairy cows is accompanied by clear physiological and metabolic shifts under various stress conditions. These physiological alterations frequently depress voluntary feed intake and induce nutrient insufficiency in lactating dairy cows. Although mid-lactation cows generally recover from the most severe negative energy balance observed in early lactation, high-yielding cows may still experience a gap between nutrient requirements and dry-matter intake due to sustained high milk production. This status further triggers oxidative stress [1], inflammation [2], and immune dysfunction [3], increasing the risk of multiple metabolic disorders and reducing farm economic returns, which greatly constrains dairy production in China. Oxidative stress arises from an imbalance between reactive oxygen species production and antioxidant defense capacity, and its importance in dairy cattle health has been extensively reviewed [4].

Due to the influence of anabolic processes during pregnancy, feed restriction measures are often adopted in dairy production. Specifically, a reduced nutrient concentration and concentrate-to-roughage ratio are used to control the newborn calf weight and limit dystocia. In clinical production, anionic salts are often supplemented in the feed to manage the dietary cation–anion difference (DCAD). However, due to the dramatic dietary changes pre- and post-calving, the continuous addition of anionic salts can increase the risk of acidosis in lactating cows. When studying the effect of DCAD on ruminant activity in periparturient dairy cows, Goff et al. [5] found that cows fed with high-cation diets were prone to metabolic alkalosis, which leads to hypocalcemia and puerperal fever, while cows fed with an acidogenic diet were susceptible to compensatory metabolic acidosis. Similarly, Caixeta et al. [6] found that an acidogenic diet reduced the incidence of hypocalcemia but increased the risk of rumen acidosis, thus negatively impacting lactation productivity and the sustainable development of the dairy industry. Although most DCAD studies have focused on the periparturient period, mid-lactation high-yield cows also face challenges related to acid–base balance and mineral homeostasis due to sustained high milk production.

Studies on the effect of the addition of anionic salts to the feed on ruminant milk production remain controversial. Moreover, studies on the evaluation of the addition of alkaline buffer and acid buffer on ruminants have not brought a unified understanding. A meta-analysis conducted by Santos et al. [7] on the effects of DCAD on production performance and health of periparturient cows revealed that an acidogenic diet provided during the periparturient period did not affect the milk yield, protein yield, milk fat metabolism, or fat yield in primiparous cows. However, in multiparous cows, reducing DCAD from +200 mEq/kg to −100 mEq/kg in the diet during the periparturient period increased total blood calcium levels (from 1.86 mM to 2.04 ± 0.05 mM), DMI (to 1.0 kg/day), and milk yield (to 1.7 kg/day) on the calving day. A separate meta-analysis by Hu and Murphy [8] further confirmed that dietary DCAD significantly affects dry-matter intake, milk production, and blood acid–base balance in lactating dairy cows. Furthermore, Matamoros et al. [9] reported that short-term feeding of sodium acetate and sodium bicarbonate increased DCAD in the feed, thus suggesting that allogenic ratios could improve the total dietary digestibility and increase milk fat yield. In addition, an in vitro study by Wu et al. [10] confirmed that the addition of rumen buffer to the feed could improve the degradation rate of rumen dry matter and neutral detergent fiber. Moreover, Qi et al. [11] found that alkaline buffer improved respiratory health and growth performance in transport calves. In lactating dairy cows, Iwaniuk et al. [12] further demonstrated that both the dietary DCAD concentration and cation source significantly affected milk production and feed efficiency. Similarly, Tucker et al. [13] reported that increasing the dietary cation–anion balance from −10 to +50 mEq/kg DM linearly improved the milk yield and feed intake in lactating Holstein cows, supporting the beneficial role of alkalogenic diets during lactation.

Applying anion and cation buffers during lactation in a scientifically and reasonably proven manner is particularly relevant in dairy production. Alkaline mineral complex (AMC) water, an alkaline solution containing several ions including Na^+^, K^+^, Zn^2+^ and Ge^4+^, among others, has anti-inflammatory, antioxidant, intestinal health- and growth-promoting effects [14]. Nevertheless, it remains unclear whether dietary supplementation with AMC water can modify the production performance, oxidative-stress status, and immune indices in high-yield mid-lactation dairy cows. Accordingly, the present study was carried out to test the hypothesis that dietary AMC supplementation would modulate the production performance and physiological status across mid-lactation dairy cows with divergent milk yield levels.

## 2. Materials and Methods

### 2.1. Ethics Committee Approval

All the procedures with animals were approved by the China Agricultural University Laboratory Animal Welfare and Animal Experimental Ethical Inspection Committee (no. AW82305202-2-5).

### 2.2. Cows, Treatments, and Management

One hundred and twenty multiparous Holstein cows were selected from the Jinyindao Farm of Beijing Shounong Livestock Development Co., Ltd., Beijing, China. Cows averaged 183 DIM (SD = 47) at the start of the experiment. Cows were fed with a total mixed ration formulated according to the dairy nutrient requirement and feeding standard (NY/T34-2004 [15]), and their milk yield averaged 40.1 kg/d. The feed ingredients and chemical composition of the diet are shown in Table 1.

Cows were separated into three control groups (CON) and three treatment groups (TRE) (20 cows per group) based on the average milk yield, i.e., an extra-high-yield group (EHYG), high-yield group (HYG), and medium- and high-yield group (MHYG). Baseline animal information, including parity, initial days in milk and the pre-experimental average milk yield for each subgroup, are presented in Table 2. Cows were fed in free stalls, and the TRE group fed a fully mixed AMC diluent. The AMC concentrate was diluted with tap water at a ratio of 1 part concentrate to 4 parts water (*v*/*v*). This diluted AMC solution was sprayed onto the total mixed ration (TMR) three times daily (morning, noon, evening) before feeding. The actual volume of diluted AMC solution provided per cow each day was determined according to preliminary feeding trials. Cows had free access to water and were milked three times per day. The AMC concentrate used in this study was provided by Beijing Jinaer Biotechnology Co., Ltd., Beijing, China. Some precise proportional values of AMC are commercially confidential. The main measurable mineral components included Na_2_HPO_4_, K_2_HPO_4_, K_2_CO_3_, and Na_2_SiO_3_. Each experimental period lasted 100 days, where the first 10 days were for adjustment to the treatment.

Daily health observations were carried out to monitor animal welfare throughout the experimental period. No painful interventions were applied in this trial. No serious adverse events were recorded during this study. Given the non-invasive nature of this feeding trial, formal humane endpoints were not predefined.

### 2.3. Experimental Sampling, Measurements and Chemical Analysis

#### 2.3.1. Feed Intake

Individual daily feed intake for each cow was recorded by automated feed intake stations. Representative samples of the feed offered were sampled. These samples were dried in forced air oven at 65 °C for 48 h, air equilibrated and weighed. Dried samples were then pooled by cow and period and were finely ground by mortar and pestle to pass through a 1 mm screen, and then retained in sealed containers for the determination of DM and starch. Feed and orts were dried at 105 °C for 48 h in a forced air oven for the determination of DM. The total starch was determined based on the thermostable α-amylase and amyloglucosidase method [17] according to the AOAC [18].

#### 2.3.2. Milk Yield and Sample Collection

Milk yield for each cow was recorded automatically via the cow identification and flow recording systems in the dairy hall. Milk samples were collected three times daily on days 0, 30, 60 and 90 according to the milking time. Milk samples were composited at a ratio of 4:3:3 (morning:noon:evening), and were preserved with potassium dichromate, then stored at 4 °C until they were analyzed for milk fat, protein and lactose percentages, somatic cell count (SCC), and milk urea nitrogen (MUN) using a near-infrared reflectance spectroscopy analyzer (Milk Scan 605, Foss Electric, Hillerød, Denmark) in the Beijing Dairy Cattle Center. Detection methods for milk fat, protein, and lactose followed the NY/T2659-2014 standard [19], for MUN followed the ZD-70-2015 standard [20], and for SCC followed the NY/T800-2004 standard [21].

#### 2.3.3. Blood Sample Collection and Analysis

On days 0, 15, 30, 45, 60 and 90, blood samples were collected from the same 10 cows within each replicate at every time point. These animals served as repeated-measures units, and the statistical model accounted for within-cow correlation among the repeated observations. Blood samples were collected via jugular venipuncture into Vacutainer tubes (Becton Dickinson, Franklin Lakes, NJ, USA) before morning feeding. Samples were collected for the analysis of hematological parameters, including the white blood cell count (WBC), neutrophil count (NEUT), lymphocyte count (LYMPH), monocyte count (MONO), eosinophil count (EO), and basophil count (BASO).

Samples were centrifuged at 3000× *g* for 15 min, and the supernatants (plasma) were collected and frozen at −20 °C until analysis for the determination of parathyroid hormone (PTH, H207), calcitonin (CT, H153-1-2), superoxide dismutase (SOD, A001-3-2), malondialdehyde (MDA, A003-1-2), catalase (CAT, A007-5-1), H_2_O_2_ (A064-5-1)_,_ alkaline phosphatase (ALP, A059-2-2), heat shock protein 70 (HSP-70, H264-2-2), NEFA (A042-1-1), BHBA (E030-1-1), glucose (GLU, F006-1-1), cholesterol (CHOL, A111-1-1), triglycerides (TG, A110-1-1), blood urea nitrogen (BUN, C013-1-1), creatinine (CRE, C011-2-1), aspartate aminotransferase (AST, C010-1-1), total protein (TP, A045-2-2), albumin (ALB, A028-2-1), alanine aminotransferase (ALT, C009-2-1), total bilirubin (TBIL, C019-1-1), immunoglobulin A (IgA, H108-1-2), immunoglobulin G (IgG, H106-1-1), immunoglobulin M (IgM, H109-1-1), interleukin-2 (IL-2, H003-1-1), interleukin-6 (IL-6, H007-1-1), tumor necrosis factor-α (TNF-α, H052-1-1) and granulocyte-macrophage colony-stimulating factor (GM-CSF, H060).

Concentrations of WBC, NEUT, LYMTH, MONO, EO and BASO were detected at the Beijing Laboratory Science and Technology Development Co., Ltd., Beijing, China. Blood cells were detected in an automatic blood cell counter (MEK6318K; Nihon Kohden, Tokyo, Japan). Routine blood indicators were detected at the Beijing Laboratory Science and Technology Development Co., Ltd., Beijing, China. Blood cells were detected in an automatic blood cell counter (Japanese photoelectric MEK6318K). Plasma concentrations of PTH, CT, CAT, H_2_O_2_, ALP, HSP-70, GLU, CHOL, TG, BUN, CRE, AST, TP, ALB, ALT, TBIL, IgA, IgG, IgM, IL-2, IL-6, TNF-α and GM-CSF were analyzed using commercial ELISA kits (Nanjing Jiancheng Biotechnology, Nanjing, China). The concentrations of SOD, MDA, BUN, BHBA, and NEFA were all determined using the corresponding assay kit (Beijing Strong Biotechnologies, Inc., Beijing, China) in an automated biochemistry analyzer (Model 7020, Hitachi Co., Tokyo, Japan).

### 2.4. Statistical Analyses

All statistical analyses were performed using IBM SPSS Statistics 26.0 (IBM Corp., Armonk, NY, USA). For longitudinal data with repeated sampling over time, a repeated-measures linear mixed model (MIXED procedure) was applied. The model included the treatment (CON vs. TRE), sampling time, and the treatment × time interaction as fixed effects, with the individual cow specified as a random effect to account for within-cow correlation among repeated measurements. For production performance parameters (dry-matter intake and milk yield), data from all 120 cows were included (*n* = 60 per treatment); for blood biomarkers, a subset of 30 cows per treatment (10 cows per replicate × 3 replicates) was used. Means and the standard error of the mean (SEM) were computed for each parameter. When a significant treatment × time interaction was detected (*p* < 0.05), post-hoc pairwise comparisons were performed to compare CON and TRE at each sampling time point. Tables report SEM and *p*-values for the treatment main effect, time main effect, and treatment × time interaction. For parameters with only the treatment *p*-value reported (dry-matter intake and milk yield), the value represents the treatment main effect derived from the same mixed model. Significance was declared at *p* < 0.05, and 0.05 ≤ *p* < 0.10 was considered a tendency.

## 3. Results

### 3.1. Feed Intake and Milk Yield

**Figure 1:**
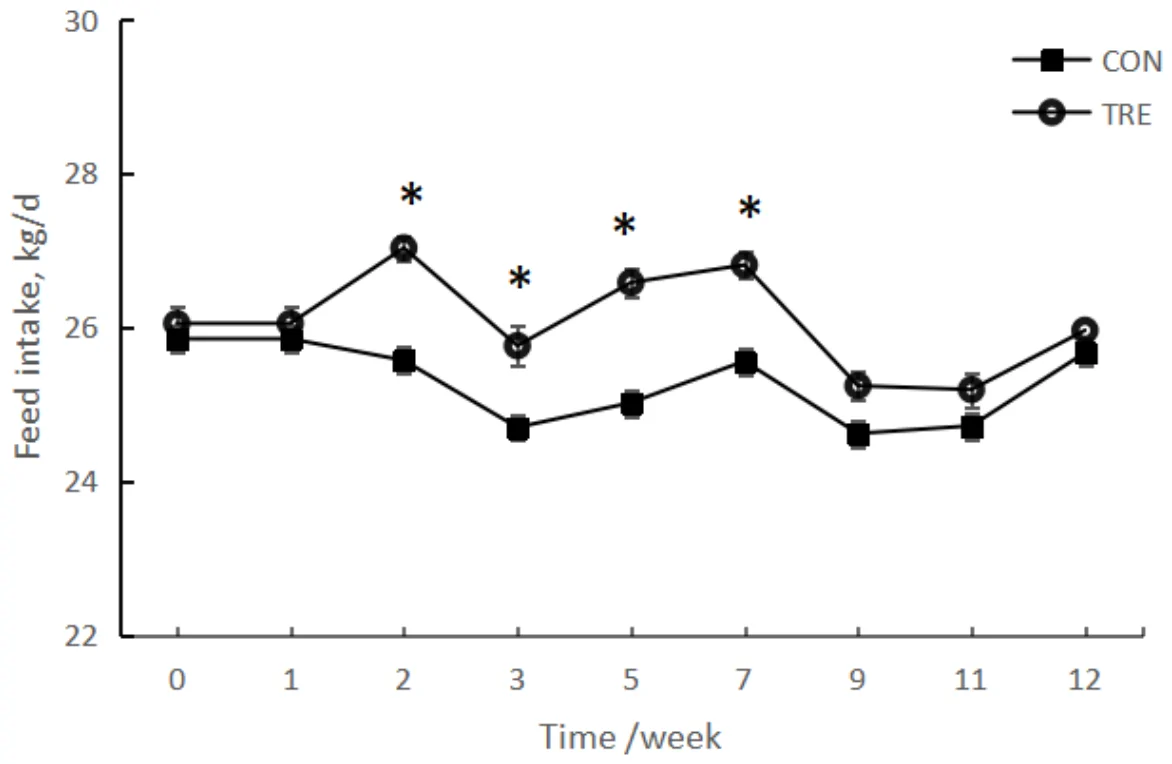
Feed intake varied with days of lactation cows feeding on AMC supplement. CON, cows feeding on TMR ration; TRE, cows feeding on TMR ration with addition of AMC diluent. For each time point, * means significantly different (*p* < 0.05).

As shown in Table 3, the average dry-matter intake (DMI) of dairy cows in the TRE group was significantly higher than that in the CON group throughout the experimental period (treatment effect, *p* < 0.05). No difference was observed in baseline DMI between TRE and CON groups before the experiment (*p* > 0.05). After AMC water supplementation to the diet, DMI in the TRE group showed a numerical increase at most sampling weeks, with significant differences detected during weeks 3–7 (*p* < 0.05, Figure 1). On week 9, DMI in the TRE group showed an increasing trend (0.05 < *p* < 0.10), whereas no significant difference was observed in week 11 (*p* > 0.05). Both groups exhibited a similar transient decrease in DMI during week 3, likely reflecting environmental or management fluctuations.

**Figure 2:**
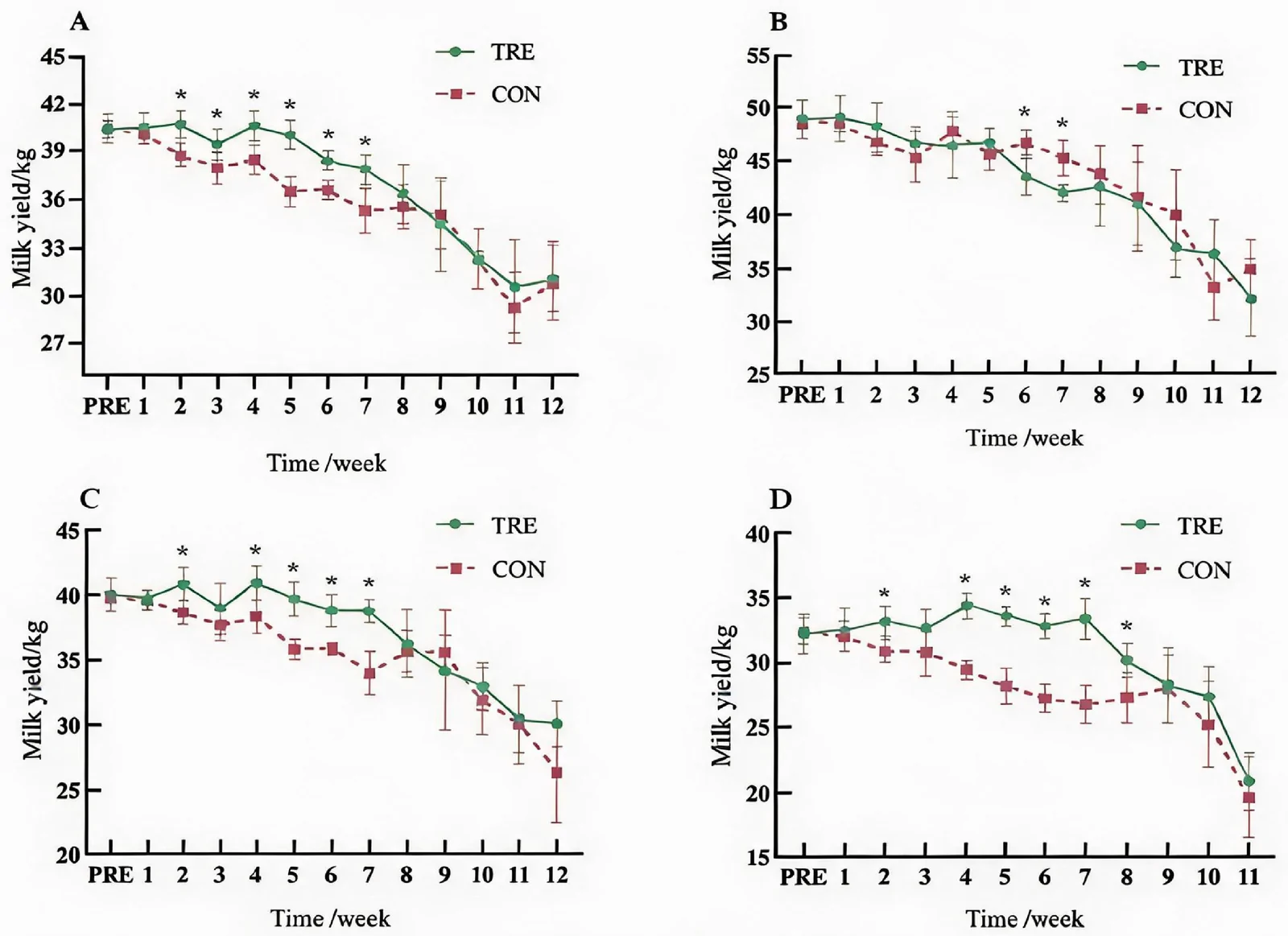
Milk yield varied with week of lactation cows feeding on AMC supplement. CON, cows feeding on TMR ration; TRE, cows feeding on TMR ration with addition of AMC diluent. (**A**) TRE. All tested cows. (**B**) EHYG. Extra-high- yield cows. (**C**) HYG. High-yield cows. (**D**) MHYG. Medium- and high-yield cows. For each time point, * means significantly different (*p* < 0.05).

The milk yield of experimental cows exhibited temporal fluctuations over the experimental period (Figure 2). Both TRE and CON groups exhibited normal temporal fluctuations in milk yield across the experimental period (Table 3). For overall cows, the total milk yield was significantly greater in the TRE group relative to the CON group during weeks 2–7 (*p* < 0.05), whereas no statistical differences were detected in the remaining sampling weeks (*p* > 0.05). When stratified by milk yield subgroups, significant subgroup specific responses were observed. For the EHYG subgroup, cows in the CON group produced a significantly higher milk yield than TRE cows during weeks 6–7 (*p* < 0.01). In the HYG subgroup, the milk yield was significantly higher for TRE cows compared with CON cows at week 2 and weeks 4–7 (*p* < 0.05), with a highly significant difference at week 7 (*p* < 0.01). For the MHYG subgroup, the milk yield did not differ significantly between TRE and CON throughout the trial (*p* > 0.05), although TRE cows exhibited a tendency toward an increased milk yield (0.05 < *p* < 0.10).

### 3.2. Milk Composition

AMC water supplementation to the diet showed no significant effect on the milk composition of lactating cows (Table 4). However, changes in milk fat content, protein content, fat–protein ratio, and lactose content were significantly correlated with time (*p* < 0.05). Interestingly, the MUN content in milk in the TRE group on day 60 was significantly higher compared to that in the CON group (*p* < 0.05), reaching 11.28 mg/dL.

### 3.3. Hormone Concentrations

No significant effect was observed on PTH levels in lactating cows after AMC water supplementation in the diet. However, the CT content on day 60 in the TRE group was significantly higher compared to that in the CON group (*p* < 0.05), reaching 2.29 pmol/L. No significant difference was found at the other time points. The CT content was significantly correlated with time (*p* < 0.05) (Table 5).

### 3.4. Glycolipid Metabolism

**Figure 3:**
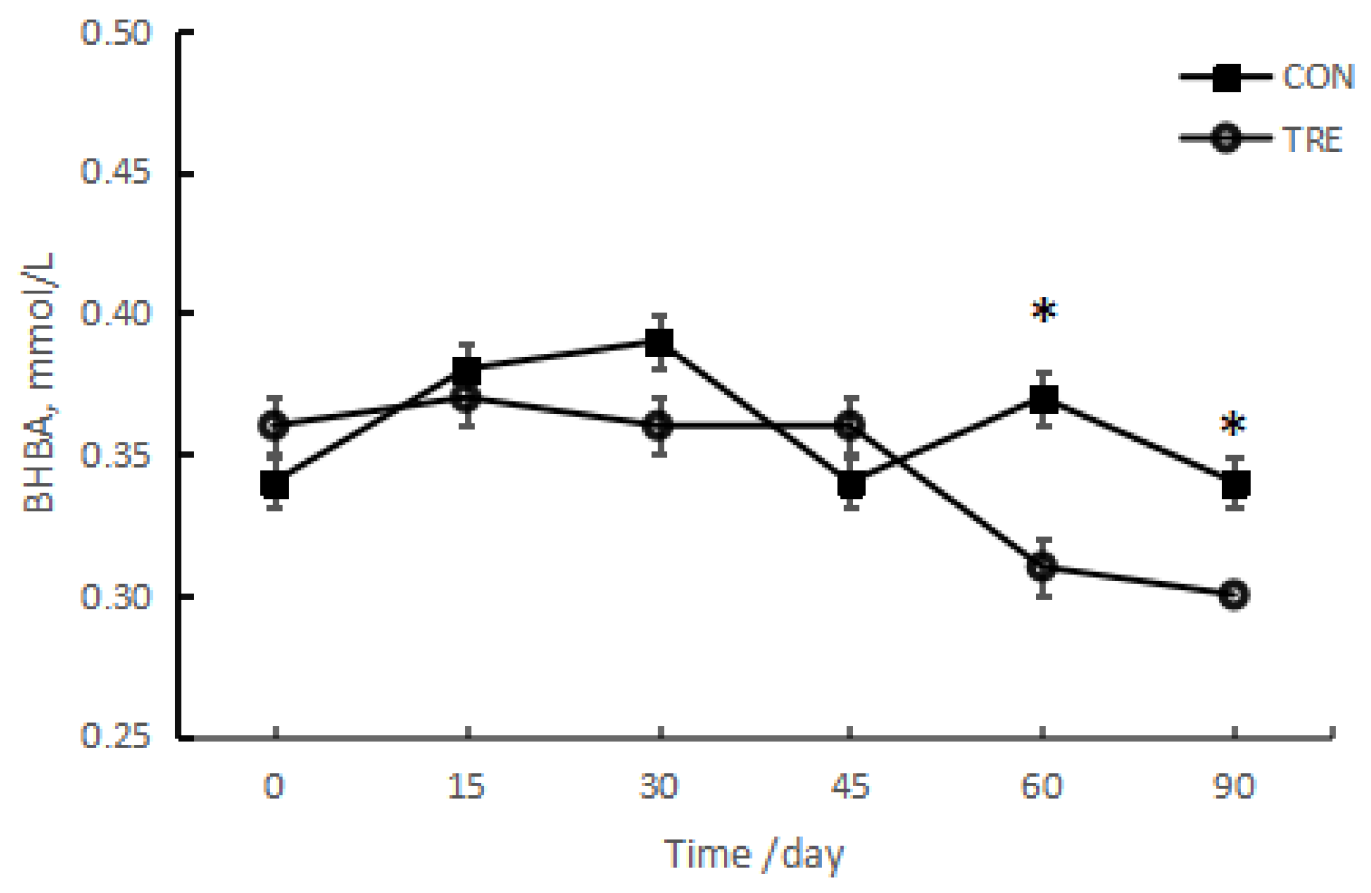
BHBA varied with days of lactation cows feeding on AMC supplement. CON, cows feeding on TMR ration; TRE, cows feeding on TMR ration with addition of AMC diluent. For each time point, * means significantly different (*p* < 0.05).

As shown in Table 6, no significant effect was observed after AMC water supplementation in the diet of glucose and lipid metabolism of tested cows, including GLU, CHOL, TG, BUN, and CRE. However, BHBA indicators were significantly decreased in cows of the TRE group (*p* < 0.05). On day 90, BHBA in the TRE group (0.30 mmol/L) was significantly lower than that in the CON group (0.34 mmol/L) (*p* < 0.01), although both values were within the normal range (Figure 3). Moreover, NEFA in the TRE group was significantly lower compared to that in the CON group (*p* < 0.01) throughout the experiment.

### 3.5. Liver Function

**Figure 4:**
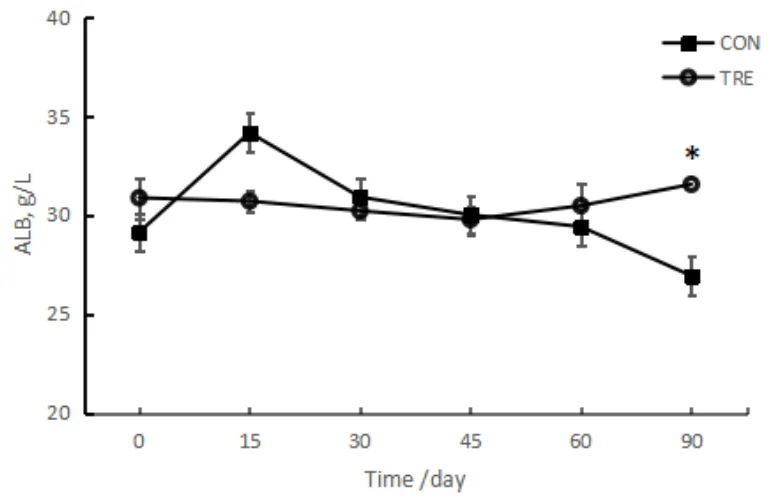
Albumin varied with days of lactation cows feeding on AMC supplement. CON, cows feeding on TMR ration; TRE, cows feeding on TMR ration with addition of AMC diluent. For each time point, * means significantly different (*p* < 0.05).

AMC water supplementation in the diet did not result in a significant impact on liver function-related indicators (AST, TP, ALB, ALT, and TBIL) in all tested cows (Table 7). However, TBIL in the TRE group was significantly higher compared to that in the CON group on day 45 (*p* < 0.05), and ALB content in the TRE group was significantly higher compared to that in the CON group on day 90 (*p* < 0.05, Figure 4).

### 3.6. Oxidant Stress

**Figure 5:**
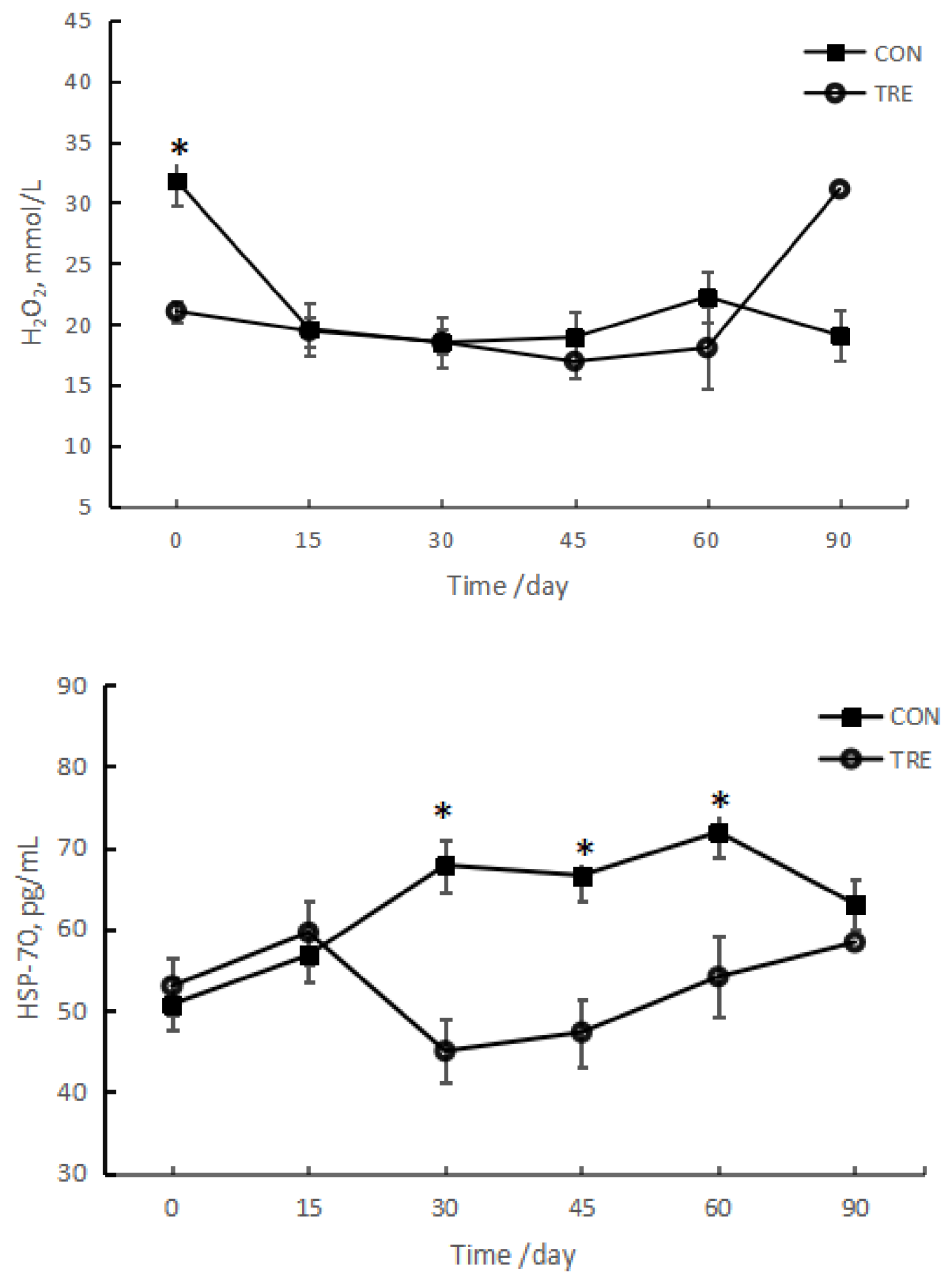
H_2_O_2_ and HSP-70 varied with days of lactation cows feeding on AMC supplement. CON, cows feeding on TMR ration; TRE, cows feeding on TMR ration with addition of AMC diluent. For each time point, * means significantly different (*p* < 0.05).

Across the experimental period, dietary AMC supplementation exerted no significant overall treatment effects on most measured oxidative stress biomarkers in cows, including SOD, MDA and CAT (Table 8). Temporal effects were detected for H_2_O_2_ and ALP concentrations (Figure 5). Although the HSP-70 concentration was significantly lower in the TRE group compared with the CON group from day 30 to day 60 (*p* < 0.05), this difference was limited to this specific time window and was not accompanied by consistent changes in other oxidative stress markers. Therefore, this single marker difference cannot be interpreted as a general improvement in the systemic oxidative status.

### 3.7. Immune Status

**Figure 6:**
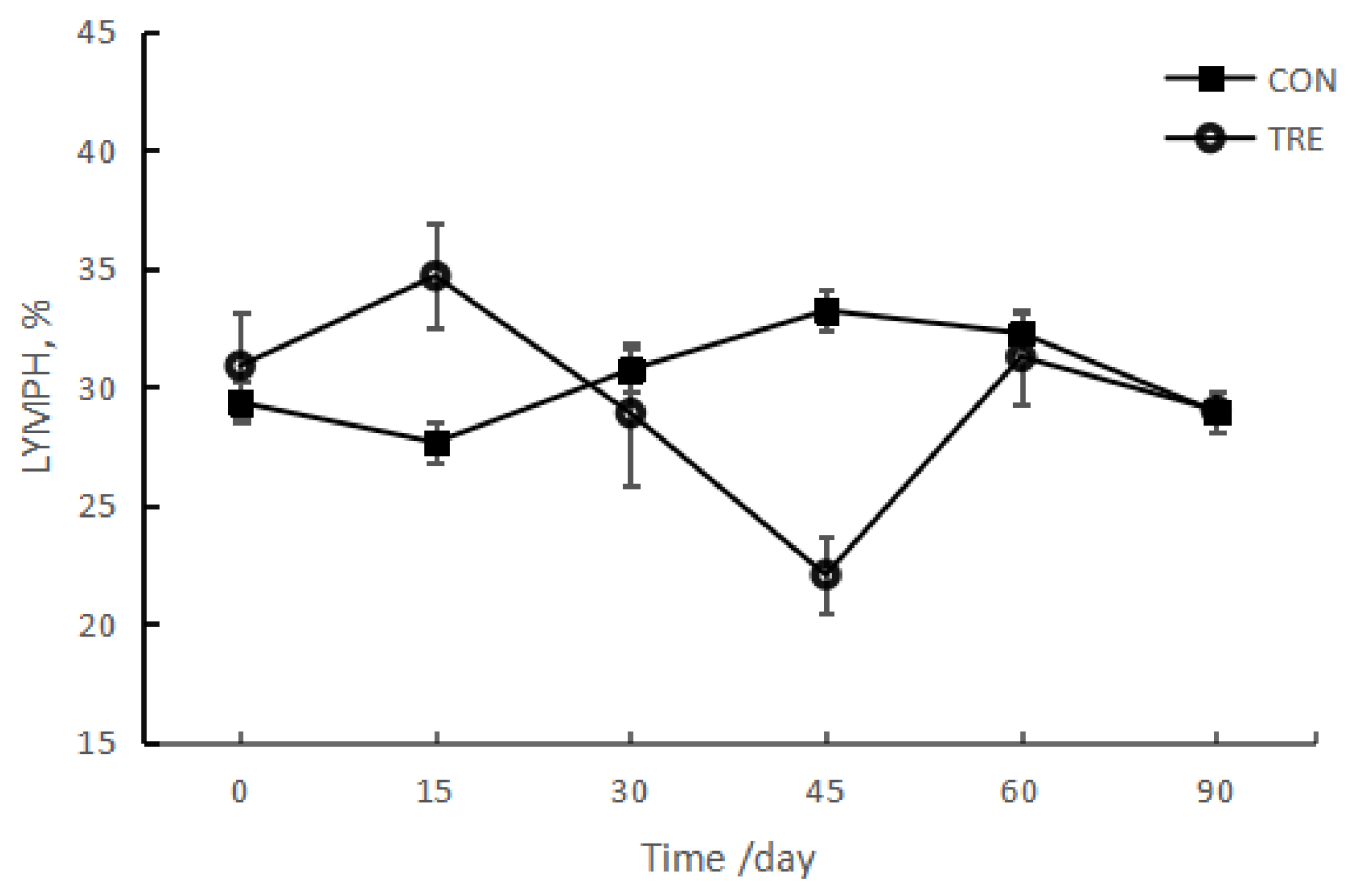
LMYPH (%) varied with days of lactation cows feeding on AMC supplement. CON, cows feeding on TMR ration; TRE, cows feeding on TMR ration with addition of AMC diluent.

No significant effect was observed on immune indicators (white blood cell count, lymphocyte ratio, and granulocyte ratio) in lactating cows after AMC water supplementation in the diet during the test period (Table 9). Of note, the absolute value of eosinophils in the CON group was significantly higher than that in the TRE group on day 90 (*p* < 0.05, Figure 6).

### 3.8. Immune Factors

**Figure 7:**
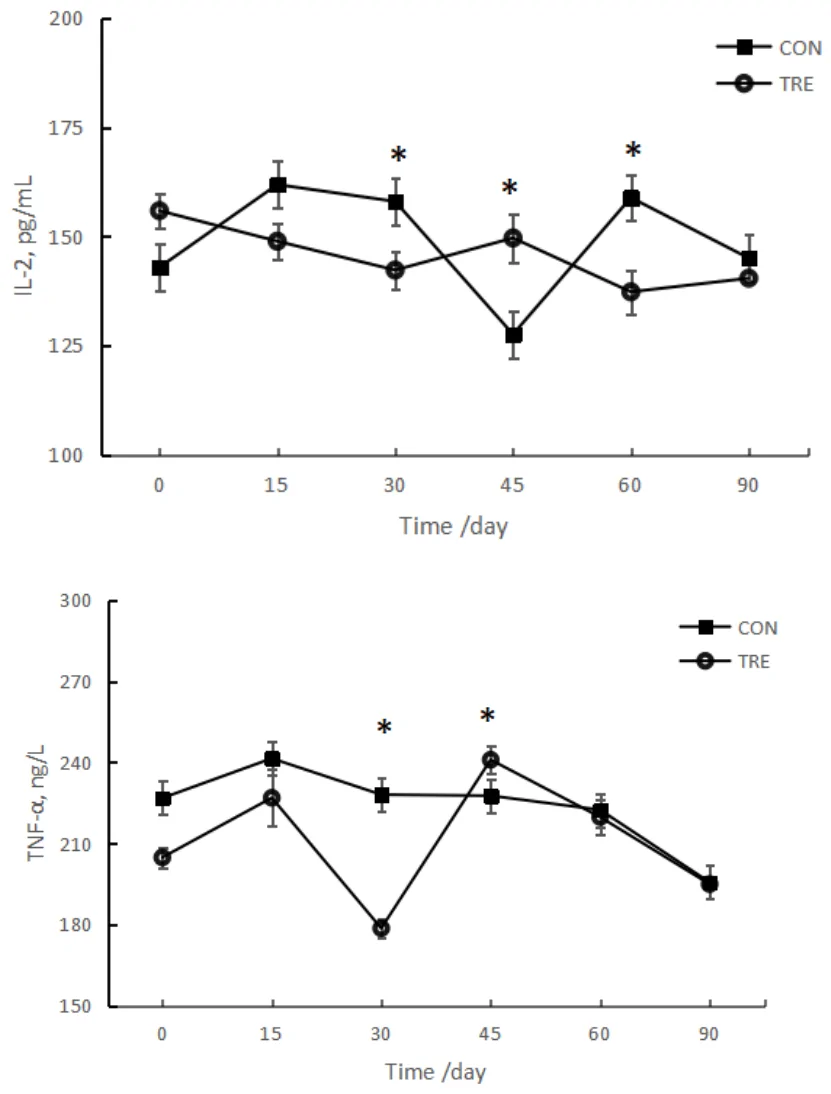
IL-2 and TNF-α varied with days of lactation cows feeding on AMC supplement. CON, cows feeding on TMR ration; TRE, cows feeding on TMR ration with addition of AMC diluent. For each time point, * means significantly different (*p* < 0.05).

Throughout the experiment, AMC supplementation significantly decreased the plasma concentrations of IgA (*p* = 0.02), IL-2 (*p* = 0.01), TNF-α (*p* = 0.01), and GM-CSF (*p* < 0.01) compared with the CON group (Table 10). No significant treatment effects were observed for IgG, IgM, or IL-6. For IL-2, a significant treatment × time interaction was detected (*p* < 0.01, Figure 7): IL-2 levels were significantly higher in the TRE group on day 45 (*p* < 0.01), but significantly lower in the TRE group on day 60 (*p* < 0.05).

## 4. Discussion

Mid-lactation is a critical production phase for high-yield dairy cows, and feeding management during this period directly influences the milk production, milk quality, reproductive performance, and productive lifespan of cows. Inadequate feeding management during mid-lactation not only reduces the milk yield and milk quality but also easily induces metabolic disorders, and eventually reduces the economic returns of dairy farms. Therefore, the feeding management level during mid-lactation has an important impact on dairy farm income.

AMC water contains essential elements such as Na, K, Zn, metasilicic acid, and rare minerals, e.g., germanium, which are critical for organ function as well as for a variety of physiological functions, including digestion and immune biosynthesis. Several studies have been carried out on the health and medical effects of mineral-rich alkaline water [22]. Studies on AMC water in humans revealed its health-promoting effects as an antioxidant, for intestine health, and for alleviating diarrhea symptoms [23,24]. In addition, AMC water has been applied in animal husbandry to alleviate heat stress in cattle, hence improving growth performance and intestinal barrier function, as well as reducing diarrhea in cattle and piglets [14]. However, it remains unclear whether AMC water can improve the health status of mid-lactation cows by alleviating oxidative stress and inflammation. In this study, AMC water addition to the diet was shown to increase the feed intake of lactating cows, although the underlying mechanism requires further investigation.

Since blood is the main carrier through which the organism maintains normal physiological functions and its internal environment, the measurement of blood biochemical indicators is often used to determine the health status of animals. In the present study, no significant differences were observed in blood routine indicators of lactating cows after the supplementation of AMC water to the diet, indicating that AMC water had no negative effect on the general health status of lactating cows.

When employing the double 3 × 3 Latin square design, Cruywagen et al. [25] found that the supplementation of sodium bicarbonate buffer to a high-concentrate diet positively impacted milk yield and milk composition in lactating cows. Moreover, when the amount added increased from 90 g/head/d to 180 g/head/d, the pH of rumen, feed conversion efficiency, and milk yield of lactating cows improved. Petrujkic et al. [26] incorporated 1% buffering mineral mixture into the concentrate diet of cows at the lactation stage and observed increases in milk yield and the contents of milk fat and protein. In the present experiment, while the mixed-model outputs indicated no significant overall treatment effect on milk yield, the HYG and MHYG subgroups receiving AMC supplementation exhibited numerically elevated milk yields. Furthermore, the milk yield of cows in the TRE group was significantly lower compared to that of cows in the CON group at certain time points. These findings suggest a numerical tendency for an increased milk yield in HYG and MHYG cows receiving AMC supplementation, although no consistent significant overall treatment effect was detected. These subgroup-level numerical tendencies should be interpreted with caution, as no consistent significant overall treatment effect was detected. Interestingly, the milk yield tended to decrease in the extra-high-yield subgroup receiving AMC supplementation. We speculate that cows with an extremely high milk output have distinct mineral requirements and metabolic profiles. Exogenous mineral inputs derived from AMC may trigger divergent physiological responses across milk yield subgroups. Potential differences in mineral tolerance among cows of varying production capacities should be acknowledged. This subgroup-specific response merits further targeted research.

Due to a negative energy balance, inflammation, oxidative stress, and immune dysfunction, cows experience metabolic and physiological changes during the lactation period. Under normal circumstances, fat metabolism can be divided into synthesis and catabolism, namely esterification of fatty acids and glycerol to form TGs, and hydrolysis of TG to produce glycerol and fatty acids. Wanapat et al. [27] found that adding a compound buffer to the diet improved nutrient digestibility and microbial nitrogen supply efficiency in lactating cows, while also reducing rumen methane production and protozoa load. BUN recovery and utilization require urea to enter the rumen, providing a valuable nitrogen source for bacterial growth, and buffering rumen acidification caused by short-chain fatty acids produced by bacteria [28]. In the present study, compared to the CON group, NEFA and BHBA levels in the TRE group decreased significantly across the experimental period, suggesting reduced fat mobilization and a more favorable energy status in lactating cows. Wei et al. [29] also found that nicotinamide lowered NEFA and BHBA levels. Thus, in the present study, AMC water supplementation in the diet significantly reduced NEFA and BHBA levels in the plasma of lactating cows within the normal range, without significantly impacting GLU, TG, BUN and CRE levels. It is worth noting that CHOL showed a tendency for increase in the TRE group (*p* = 0.05), which warrants further investigation.

In addition, the GLU content in both the CON and TRE groups decreased significantly over time, while the CHOL and BUN contents increased, indicating a strong correlation with time. It has been shown that subacute rumen acidosis can cause inflammatory damage to the liver in ruminants [30]. Herein, AMC water supplementation to the diet had no significant impact on overall liver function indicators (AST, ALT, and TBIL), although TP showed a tendency for decrease in the TRE group. Specifically, TBIL was found to be significantly increased only on day 45, while ALB was found to be significantly increased on day 90 in the TRE group (significant treatment × time interaction, *p* = 0.01). However, these two indicators were associated with many factors, and significant differences were observed only at certain time points, suggesting that the impact of AMC water supplementation might last for only a relatively short term, which may be caused by environmental factors.

Oxidative stress and immune indexes directly reflect whether there is a balance in free radical production and scavenging in lactating dairy cows. Oxidative stress is induced by an increased production of free radicals and reactive oxygen species, which decreases antioxidant defenses and causes damage to biomolecules and may lead to the emergence of metabolic and physiological disorders [31]. Oxidative stress further contributes to immune function imbalance during lactation in dairy cows [32]. Among antioxidant enzymes, CAT, SOD, and ALP have been shown to scavenge superoxide anion free radicals, while lipid peroxidation usually results in the production of MDA, which is indicative of the degree of oxidative damage. These parameters are widely recognized as key biomarkers for evaluating oxidative status in lactating dairy cows [33]. Herein, AMC water supplementation did not result in a significant overall treatment effect on antioxidant and peroxide indexes in lactating cows, including CAT, SOD, ALP, and MDA, suggesting no long-term antioxidant effects of AMC water on lactating cows. However, H_2_O_2_ and ALP showed significant time effects, and H_2_O_2_ exhibited a significant treatment × time interaction, indicating that these parameters varied with the experimental period regardless of treatment.

Furthermore, the HSP-70 index is indicative of the level of heat stress in lactating cows, and an increase in HSP-70 levels will have a negative impact on the health and production performance of cows. Herein, AMC water supplementation in the diet significantly reduced HSP-70 in lactating cows from day 30 to day 60 (significant treatment × time interaction, *p* < 0.01), which coincided with summer in the Northern Hemisphere. This suggests that AMC water supplementation in the diet might alleviate heat stress in cows during the hot summer period. Similar thermoprotective effects have been reported for other dietary additives: betaine supplementation reduced HSP-70 concentrations and improved milk performance in heat-stressed dairy cows [34], and Lonicera japonica extract enhanced the antioxidant status and immune function in heat-stressed mid-lactation dairy cows [35]. Notably, on day 90, this mitigating effect was not significant, which might be related to decreased ambient temperature. However, this single-marker difference cannot be interpreted as a general improvement in the systemic oxidative status, as other oxidative-stress biomarkers did not show consistent changes. Heat stress is known to impair production performance and induce oxidative challenge in lactating dairy cows [36].

Regarding immune factors, AMC supplementation significantly decreased plasma concentrations of IgA (*p* = 0.02), IL-2 (*p* = 0.01), TNF-α (*p* = 0.01), and GM-CSF (*p* < 0.01) compared with the CON group. These reductions in pro-inflammatory cytokines (TNF-α, GM-CSF, and IL-2) and IgA suggest that AMC supplementation may attenuate inflammatory responses in mid-lactation dairy cows. TNF-α and GM-CSF are key pro-inflammatory mediators, and their down-regulation is generally associated with reduced systemic inflammation. However, IgG and IgM were not affected by treatment, and IL-6 showed no significant difference, indicating that the immunomodulatory effect of AMC was selective rather than comprehensive. It should be noted that a reduced IgA concentration may also reflect decreased antigenic stimulation rather than impaired immune function, and the clinical significance of these changes requires further investigation.

The present study has several limitations. First, ruminal pH and blood acid–base balance indicators were not measured; therefore, firm conclusions cannot be drawn about whether AMC supplementation modulates systemic acid–base homeostasis. Second, some trace-element proportions within the AMC additive are commercially confidential, which limits the reproducibility of the present experiment. Third, dietary treatments were allocated at the pen level, giving rise to a potential risk of pseudoreplication. Fourth, several physiological differences between groups were detected only at discrete sampling time points, rather than showing consistent overall treatment responses across the experimental period. Therefore, further investigations are needed to verify the biological effects of AMC supplementation in mid-lactation dairy cows.

## 5. Conclusions

In this study, dietary AMC supplementation reduced plasma NEFA and BHBA concentrations in mid-lactation dairy cows. Several physiological indices differed only at discrete sampling time points, with no consistent overall treatment-related effects. Variable physiological responses were observed across subgroups with different milk yield levels. These findings offer preliminary reference information for applying alkaline mineral complex additives in mid-lactation dairy-cow production. Additional studies are needed to validate its practical application potential under commercial farm conditions.

## Figures and Tables

**Table 1:** Ingredients and chemical composition of the total mixed ration.

Ingredient	% of DM	Chemical Composition (Analyzed) ^2^	Level
Alfalfa hay	12.80	CP, % DM	16.70
Oat grass	3.54	NDF, % DM	29.30
Alfalfa silage	3.87	ADF, % DM	18.20
Whole corn silage	26.32	EE, % DM	5.10
Steam-flaked corn	17.03	ASH, % DM	6.70
Corn	12.32	Ca, % DM	0.70
Whole cottonseed	5.46	P, % DM	0.30
Extruded soybean meal	4.45	Starch, % DM	24.52
Wheat bran	1.22	NE_L_, MJ/kg	7.24
Soybean hull	0.45		
DDGS	1.12		
Dried beet pellet	7.67		
sugarcane molasses	1.14		
Limestone	0.31		
NaHCO_3_	0.56		
NaCl	0.23		
Fat powder	0.45		
Premix ^1^	1.06		
Total	100		

^1^ The premix was formulated and prepared by the experimental farm. ^2^ The chemical composition of the diets was determined according to AOAC (1990) [16].

**Table 2:** Baseline characteristics of cows allocated to the control (CON) and AMC-supplemented (TRE) groups.

Item	Treatment	N	Parity	Days in Milk, d	Milk Yield, kg/d
Total	CON	60	2.67 ± 0.86	183.24 ± 47.36	39.88 ± 6.57
	TRE	60	2.67 ± 0.86	182.76 ± 47.87	40.33 ± 6.99
EHYG ^1^	CON	20	2.57 ± 0.54	127.71 ± 7.65	47.95 ± 0.83
	TRE	20	2.57 ± 0.79	127.43 ± 6.29	48.61 ± 1.53
HYG ^2^	CON	20	2.29 ± 0.95	181.86 ± 7.65	39.23 ± 1.23
	TRE	20	2.43 ± 0.98	180.71 ± 8.86	40.16 ± 1.36
MHYG ^3^	CON	20	3.14 ± 0.90	240.14 ± 14.05	32.46 ± 1.06
	TRE	20	3.00 ± 0.82	240.14 ± 10.04	32.23 ± 1.49

^1^ EHYG. Extra-high-yield cows. ^2^ HYG. High-yield cows. ^3^ MHYG. Medium- and high-yield cows.

**Table 3:** Feed intake and milk yield of lactation cows feeding on AMC supplementation.

Item	Treatment ^1^		SEM	*p*-Value
	CON	TRE		
N	60	60		
Feed intake, kg/d	25.21	26.08	0.21	0.02
Milk yield, kg/d	38.93	40.20	1.05	0.41

^1^ CON, cows feeding on TMR ration; TRE, cows feeding on TMR ration with addition of AMC diluent. Values are means across all sampling time points.

**Table 4:** Milk composition of lactation cows feeding on AMC supplementation.

Item ^2^	Treatment ^1^		SEM	*p*-Value		
	CON	TRE		Treatment	Time	Interaction
N	60	60				
Milk fat, %	4.31	4.35	1.11	0.82	0.22	0.28
Milk protein, %	3.13	3.18	0.27	0.20	<0.01	0.82
Milk lactose, %	5.22	5.25	0.21	0.30	<0.01	0.69
SCC, 10^4^/mL	28.39	28.25	74.37	0.97	0.53	0.40
MUN, mg/dL	12.90	13.82	3.48	0.06	<0.01	0.87

^1^ CON, cows feeding on TMR ration; TRE, cows feeding on TMR ration with addition of AMC diluent. ^2^ SCC, somatic cell count; MUN, milk urea nitrogen. Values are means across all sampling time points.

**Table 5:** Hormone concentrations of lactation cows feeding on AMC supplementation.

Item ^2^	Treatment ^1^		SEM	*p*-Value		
	CON	TRE		Treatment	Time	Interaction
N	30	30				
PTH, pg/mL	16.92	17.66	0.24	0.11	0.22	0.22
CT, pmol/L	2.55	2.51	0.06	0.53	0.01	0.14

^1^ CON, cows feeding on TMR ration; TRE, cows feeding on TMR ration with addition of AMC diluent. ^2^ PTH, parathyroid hormone; CT, calcitonin Values are means across all sampling time points.

**Table 6:** Glycolipid metabolism of lactation cows feeding on AMC supplementation.

Item ^2^	Treatment ^1^		SEM	*p*-Value		
	CON	TRE		Treatment	Time	Interaction
N	30	30				
NEFA, μmol/L	40.25	36.07	0.63	<0.01	<0.01	0.16
BHBA, mmol/L	0.36	0.34	0	0.03	<0.01	0.02
GLU, mmol/L	3.89	3.78	0.04	0.12	0.01	0.27
CHOL, mmol/L	6.28	6.84	0.15	0.05	0.01	0.76
TG, mmol/L	0.15	0.14	0	0.32	0.33	0.67
BUN, mmol/L	4.83	5.04	0.11	0.27	0.01	0.20
CRE, μmol/L	67.89	65.31	0.82	0.11	0.42	0.51

^1^ CON, cows feeding on TMR ration; TRE, cows feeding on TMR ration with addition of AMC diluent. ^2^ GLU, glucose; CHOL, cholesterol; TG, triglycerides; BUN, blood urea nitrogen; CRE, creatinine. Values are means across all sampling time points.

**Table 7:** Liver function of lactation cows feeding on AMC supplementation.

Item ^2^	Treatment ^1^		SEM	*p*-Value		
	CON	TRE		Treatment	Time	Interaction
N	30	30				
AST, μ/L	81.58	75.64	3.70	0.40	0.31	0.79
TP, g/L	75.59	73.25	0.57	0.05	0.58	0.83
ALB, g/L	29.91	30.63	0.33	0.31	0.05	0.01
ALT, U/L	24.16	25.53	0.88	0.52	0.01	0.49
TBIL, μmol/L	7.01	6.72	0.14	0.20	<0.01	0.14

^1^ CON, cows feeding on TMR ration; TRE, cows feeding on TMR ration with addition of AMC diluent. ^2^ AST, aspartate aminotransferase; TP, total protein; ALB, albumin; ALT, alanine aminotransferase; TBIL, total bilirubin. Values are means across all sampling time points.

**Table 8:** Oxidant stress of lactation cows feeding on AMC supplementation.

Item ^2^	Treatment ^1^		SEM	*p*-Value		
	CON	TRE		Treatment	Time	Interaction
N	30	30				
SOD, U/mL	47.49	47.48	0.90	0.95	0.01	0.13
MDA, nmol/mL	1.47	1.48	0.02	0.77	0.90	0.86
CAT, U/mL	13.00	13.11	0.42	0.92	0.68	0.71
H_2_O_2,_ mmol/L	21.41	21.18	0.89	0.91	<0.01	<0.01
ALP, U/L	37.08	40.34	1.57	0.21	<0.01	0.50
HSP-70, pg/mL	59.60	56.10	1.59	0.20	0.25	<0.01

^1^ CON, cows feeding on TMR ration; TRE, cows feeding on TMR ration with addition of AMC diluent. ^2^ SOD, superoxide dismutase, MDA, malondialdehyde; CAT, catalase; ALP, alkaline phosphatase; HSP-70, heat shock protein 70. Values are means across all sampling time points.

**Table 9:** Immune status of lactation cows feeding on AMC supplementation.

Item ^2^	Treatment ^1^		SEM	*p*-Value		
	CON	TRE		Treatment	Time	Interaction
N	30	30				
WBC, 10^9^	7.97	8.23	0.24	0.58	0.11	0.75
LYMPH, %	31.00	29.14	0.96	0.19	0.95	0.02
MONO, %	16.10	17.15	0.48	0.38	0.73	0.97
EO, %	2.53	2.09	0.18	0.22	0.24	0.47
BASO, %	0.81	0.73	0.04	0.27	0.94	0.6
NEUT, 10^9^	3.97	4.22	0.17	0.41	0.38	0.63
LYMPH, 10^9^	2.46	2.40	0.11	0.65	0.28	0.12
MONO, 10^9^	1.28	1.38	0.05	0.29	0.24	0.59
EO, 10^9^	0.20	0.17	0.02	0.46	0.37	0.34
BASO, 10^9^	0.06	0.06	0	0.76	0.26	0.44

^1^ CON, cows feeding on TMR ration; TRE, cows feeding on TMR ration with addition of AMC diluent. ^2^ WBC, white blood cell count; NEUT, neutrophil count; LYMTH, lymphocyte count; MONO, monocyte count; EO, eosinophil count; BASO, basophil count. Values are means across all sampling time points.

**Table 10:** Immune factors of lactation cows feeding on AMC supplementation.

Item ^2^	Treatment ^1^		SEM	*p*-Value		
	CON	TRE		Treatment	Time	Interaction
N	30	30				
IgA, μg/mL	528.99	497.57	7.49	0.02	0.09	0.12
IgG, mg/mL	7.98	8.18	0.08	0.18	0.03	0.12
IgM, mg/mL	1.32	1.29	0.01	0.32	0.78	0.42
IL-2, pg/mL	149.01	145.70	1.87	0.01	0.04	<0.01
IL-6, pg/mL	356.49	355.71	4.13	0.92	0.33	0.13
TNF-α, pg/mL	223.64	210.97	4.76	0.01	<0.01	0.01
GM-CSF, pg/mL	80.52	70.22	1.64	<0.01	0.82	0.47

^1^ CON, cows feeding on TMR ration; TRE, cows feeding on TMR ration with addition of AMC diluent. ^2^ IgA, immunoglobulin A; IgG, immunoglobulin G; IgM, immunoglobulin M; IL-2, interleukin-2; IL-6, interleukin-6; TNF-α, tumor necrosis factor-α; GM-CSF, granulocyte-macrophage colony-stimulating factor. Values are means across all sampling time points.

## Data Availability

All raw data and sequencing information can be requested by contacting the author Zhanshan Yang (zsyang301@163.com).

## Abbreviations

The following abbreviations are used in this manuscript:

AMC	Alkaline mineral complex
EHYG	Extra-high-yield milk production group
MHYG	Medium- and high-yield milk production group
HYG	High-yield milk production group
DCAD	Dietary cation–anion difference
CON	Control
TMR	Total mixed ration
SCC	Somatic cell count
MUN	Milk urea nitrogen
BUM	Blood urea nitrogen
IgA	Immunoglobulin A
IgG	Immunoglobulin G
IgM	Immunoglobulin M
IL-2	Interleukin 2
IL-6	Interleukin 6
TNF-α	Tumor necrosis factor-α
GM-CSF	Granulocyte-macrophage colony-stimulating factor
SOD	Superoxide dismutase
T-AOC	Total antioxidant capacity
GSH Px	Glutathione peroxidase
MDA	Malondialdehyde
PTH	Parathyroid hormone
CT	Calcitonin
ELISA	Enzyme-linked immunosorbent assay
GLU	Glucose
TG	Triglyceride
TP	Total protein
ALB	Albumin
AST	Aspartate aminotransferase
ALT	Alanine aminotransferase
ALP	Alkaline phosphatase
TBIL	Total bilirubin
CRE	creatinine
CHOL	Total cholesterol
WBC	White blood cell count
NEUT	Neutrophil ratio
LYMPH	Lymphocyte ratio
MONO	Monocyte
EO	Eosinophil ratio
BASO	Basophils
NEUT	Neutrophil count
LYMPH	Lymphocyte count
